# Study on the Color Compensation Effect of Composite Orange-Red Quantum Dots in WLED Application

**DOI:** 10.1186/s11671-020-03350-9

**Published:** 2020-05-24

**Authors:** Xiaoyue Hu, Yangyang Xie, Chong Geng, Shu Xu, Wengang Bi

**Affiliations:** Tianjin Key Laboratory of Electronic Materials and Devices, School of Electronics and Information Engineering, Hebei University of Technology, 5340 Xiping Road, Tianjin, 300401 People’s Republic of China

**Keywords:** Quantum dots, Spectral control, Lifetime, Quantum efficiency, Energy transfer

## Abstract

Quantum dots (QDs) as emerging light-converting materials show the advantage of enhancing color quality of white light-emitting diode (WLED). However, WLEDs employing narrow-emitting monochromic QDs usually present unsatisfactory color rendering in the orange region. Herein, composite orange-red QDs (composite-QDs) are developed through mixing CdSe/ZnS-based orange QDs (O-QDs) and red QDs (R-QDs) to compensate the orange-red light for WLEDs. We investigated the effect of self-absorption and fluorescence resonance energy transfer (FRET) process in composite-QDs on the spectral controllability and fluorescent quenching in WLEDs. The concentration and donor/acceptor ratios were also taken into account to analyze the FRET efficiency and help identify suitable composite-QDs for color compensation in the orange-red light region. As the result, the optimized composite-QDs effectively improve the color rendering index of the WLED compared with monochromatic QDs.

## Introduction

Light-emitting diodes (LEDs) have attracted significant research interests in solid-state lighting applications due to their high efficiency, long lifetime, low-power consumption, fast response time, and high reliability [1–6]. WLEDs are usually fabricated by packaging yellow-, green-, and red-emitting phosphors with blue-LED chips [7–9]. The full-spectrum WLEDs employ composite phosphors with a high proportion of red phosphor [10]. However, classical red phosphors have a broad emission that causes lumen loss in the red-light-emitting region because the human eye is insensitive to the wavelength longer than 650 nm [11].

Recently, quantum dots (QDs) have been employed to fabricate high-quality WLEDs. Compared to classical phosphors, QDs have unique optical properties, such as size-dependent wavelength tunability, high photoluminescence quantum yield, and strong absorption [12–17]. Because of the narrow-emitting characteristics at red-light region, the red-emitting QDs are particularly useful for inhibiting the abovementioned lumen loss and improving color rendering index (CRI) of WLEDs [18, 19]. Therefore, using QDs to compensate for the orange-red region has become an effective measure for enhancing the color quality of WLEDs [20]. Generally, QD-based WLEDs (QWLEDs) can be divided into two categories by mixing monochromic or polychromatic QDs in the LEDs [20–23]. For example, Xie et al. used red-emitting CdSe/CdS/ZnS QDs to replace classical red phosphor with LuAG:Ce green phosphor to fabricate high-performance WLED [24]. Li et al. fabricated QWLEDs by integrating a mixture of red, yellow, and green light-emissive CdZnS/ZnSe QDs on the blue-emitting GaN LED chip, which exhibited a CRI of 85.2 and correlated color temperature (CCT) of 4072 K [25].

To date, the full-spectrum QWLEDs for lighting application are commonly developed by incorporating broad-emitting green-yellow phosphors and narrow-emitting monochromatic red QDs [24]. These QWLEDs present superb spectral continuity in the green-yellow region but a clear valley in the orange-red region. Theoretically, the composite-QDs made of several monochromic QDs in the orange-red region are capable to fill the valley and further improve the spectral continuity of QWLEDs. However, it is difficult to regulate the spectra of the composite-QDs because of the self-absorption and fluorescence resonance energy transfer (FRET) process among polychromatic QDs [26]. Therefore, although the color property of the QWLEDs have been investigated by manipulating the peak position and broadness of the monochromatic red QDs, the composite orange-red QDs (composite-QDs) have not been studied in WLEDs on account of the self-absorption and FRET process.

Herein, composite-QDs were studied to enhance the spectral continuity and color quality of the orange-red light-emitting region for QWLEDs. We prepared CdSe/ZnS-based orange QDs (O-QDs) and red QDs (R-QDs) with different full width at half maximum (FWHM) as the component of the composite-QDs. The FRET in the composite-QDs was studied by considering the effects of concentration and proportion of composite-QDs. The results were used to optimize the quantum efficiency (QE) and spectral controllability of the composite-QDs. Moreover, the composite-QDs were used with LuAG:Ce green phosphor in blue LEDs to form QWLEDs. The as-prepared QWLEDs exhibit enhanced color quality with a more balanced full spectrum in the orange-red region.

## Methods

### Materials and Chemicals

1-Octadecene (ODE, 90%), sulfur (S, 98.5%), trioctylphosphine (TOP, 85%), and stearic acid (98%) were purchased from TCI (Shanghai). Cadmium stearate (Cd(St)_2_) was purchased from Shanghai Debo Chemical Technology Co., Ltd. Selenium powder (Se, 325 mesh, 99.5%) was purchased from Alfa Aesar (China). Zinc acetate (Zn(Ac)_2_, 99.5%) was purchased from Shanghai Titan Scientific Co., Ltd. Ethanol and dimethylbenzene were purchased from Tianjin Damao Chemical Reagent Co., Ltd. Silicone resin (Dow Corning-6662) was from Shineon Co., Ltd. Other materials are shown in the manuscript. All chemicals were used directly without any further purification unless otherwise stated.

### Synthesis of O-QDs

The synthetic procedure was based on the report in the literature [27]. Cd(St)_2_ (2 mmol) and stearic acid (0.2 mmol) were loaded into a 50-mL three-neck flask with 10 mL of ODE. After stirring with nitrogen bubbling, the solution was heated to 270 °C. Then 0.5 mL of TOP-Se (2 mmol of Se powder dissolved in 1 mL of TOP) was rapidly injected into the flask and maintained at 270 °C for 2 min. Afterward, 0.5 mL of TOP-S (4 mmol of S powder dissolved in 2 mL of TOP, stirred well) was rapidly injected into the flask and maintained at 270 °C for 40 min, and then the flask was cooled to 30 °C. Cd(St)_2_ (0.75 mmol), Zn(Ac)_2_ (2.25 mmol), and 5 mL of ODE were added into the above solution. After stirring with nitrogen bubbling, the flask was heated to 160 °C. 1.5 mL of TOP-S was slowly injected into the flask and maintained at 160 °C for 4 h, and then the flask was cooled to room temperature. After a centrifuged purification procedure with ethanol, the as-prepared CdSe/ZnS QDs were dispersed in 10 mL of dimethylbenzene for further use.

### Synthesis of R-QDs

The synthetic procedure was similar to that of O-QDs except for the following two points. The heating temperature adjusted from 270 to 300 °C. And the second added Cd(St)_2_ is 1 mmol together with Zn(Ac)_2_ (3 mmol).

### Preparation of O-QD and R-QD Silicone Gel Thin Films

Different weights of R-QDs were homogeneously mixed into the same volume silicone gels to build the different concentration R-QD gels (0.05, 0.1, 0.2, 0.4, 0.8, 2, 4, and 10 mg/mL). Then, different concentrations of R-QD gels with the same volume were added into the same type of molds and removed the bubbles. Finally, the R-QDs silicone composite thin films were built by curing at 150 °C for 60 min. The O-QDs silicone thin films are fabricated by the same process with different concentrations (0.05, 0.1, 0.2, 0.4, 0.8, 2, 4, 10, and 14 mg/mL).

### Preparation of composite-QD Silicone Gel Thin Films with Different Weight Ratios of O-QDs to R-QDs

The composite-QD silicone gel with different weight ratios of O-QDs to R-QDs (10:1, 5:1, 5:2, and 5:4) were prepared by homogeneously mixing the as-prepared O-QD gel (10 mg/mL) and R-QD gel (2 mg/mL) with different volume ratios (2:1, 1:1, 1:2, and 1:4). Then, the different concentrations of composite-QD gels were added into the same type of mold and removed the bubbles. Finally, the composite-QD silicone gel thin films were built by curing at 150 °C for 1 h.

### Preparation of composite-QD Silicone Gel Thin Films with Different Concentrations

With the same weight ratio of O-QDs to R-QDs (10:1), the composite-QDs were mixed into the different volumes of silicone gels to form composite-QD gels with different concentrations (0.35, 0.5, 0.75, 1, 1.5, and 3 mg/mL). Then, the as-prepared composite-QD gels were added into the same type of molds and removed the bubbles. Finally, the composite-QD silicone gel thin films with different composite-QD concentrations were built by curing at 150 °C for 1 h.

### Fabrication of WLEDs

The LED chips (typical 2835 lead frame package) with the emission peak at 450 nm were used for the fabrication of WLEDs.

Green-emitting LuAG:Ce phosphor, O-QDs (10 mg/mL), R-QDs (2 mg/mL), or composite O-R QDs (weight ratio 10:1) were mixed homogeneously with silicone gel (Dow Corning 6662, A:B = 1:4), and the mixture was degassed under vacuum. With a common packaging method based on silicone gel, the four different WLEDs were developed with LuAG:Ce phosphor, phosphor and O-QDs, phosphor and R-QDs, and phosphor and composite-QDs, respectively. Finally, the above WLEDs were cured by curing at 150 °C for 1 h.

### Measurement and Characterization

Photoluminescence (PL) was recorded on an Ideaoptics FX2000-EX PL spectrometer. Transmission electron spectroscopy (TEM) was performed on a FEI Tecnai G2 Spirit TWIN transmission electron microscope operating at 100 kV. The quantum efficiency (QE) measurements were carried out on an OceanOptics QEpro QY test system under 365 nm blue laser irradiation. The luminous efficiency and optical power were recorded on an EVERFINE ATA-1000 LED automatic temperature control photoelectric analysis and measurement system. UV-Vis absorption was measured by using a Persee T6 UV-Vis spectrometer. The excitation spectra and time-resolved PL spectroscopy (TRPL) have been measured by an Edinburgh FLS920 fluorescence spectrometer.

## Results and Discussion

**Fig. 1:**
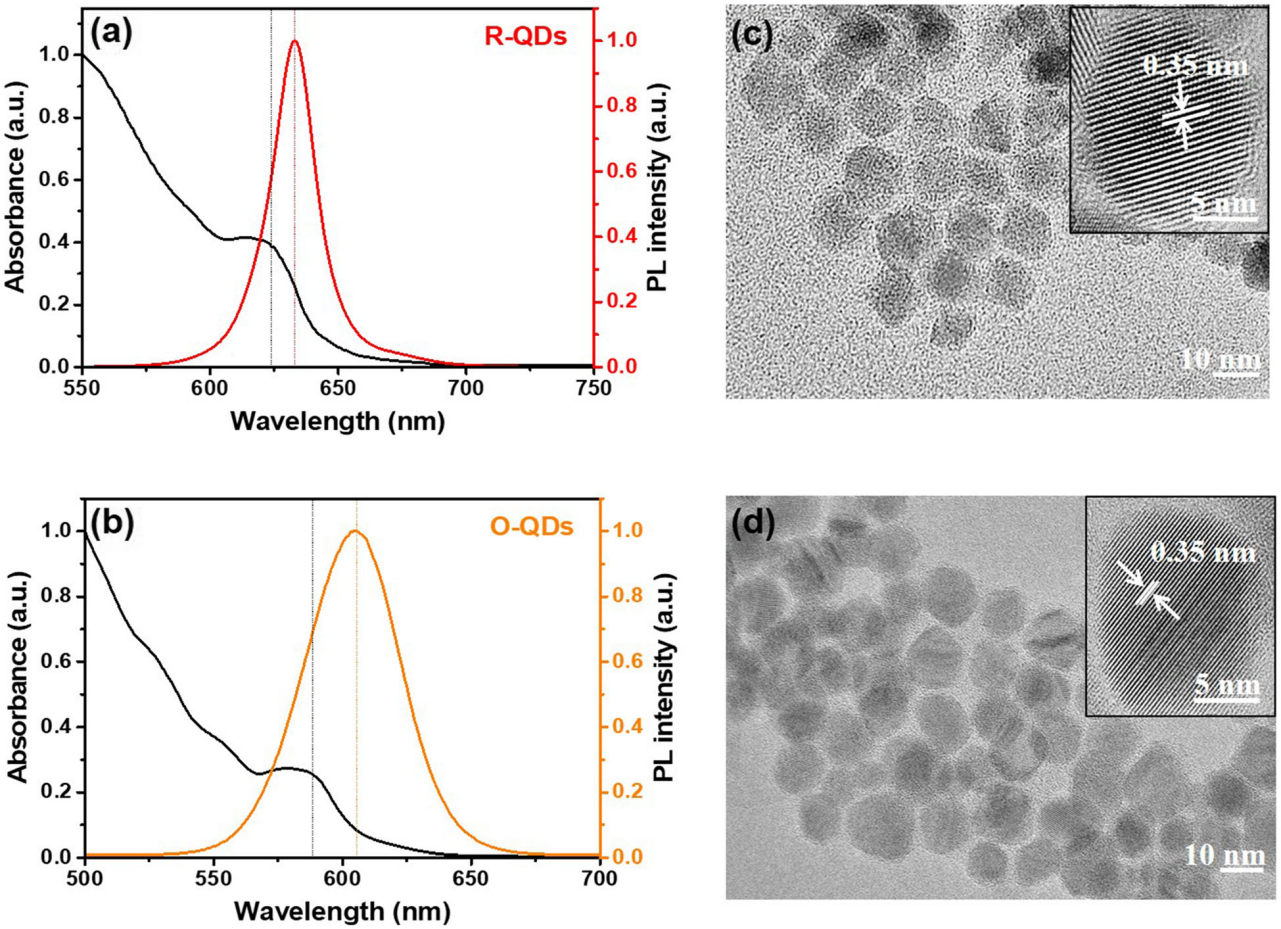
PL and UV spectra of R-QDs (**a**) and O-QDs (**b**). TEM images of R-QDs (**c**) and O-QDs (**d**)

The optical properties of the two monochromic QDs were firstly studied. Figure 1a and b show the photoluminescence (PL) and absorption spectra of the R-QDs and O-QDs. The FWHM of the R-QDs and O-QDs is about 20.6 and 43 nm, respectively. The positions of dotted lines indicate the PL and absorption peaks. As shown in TEM images, the R-QDs and O-QDs exhibit a cubic morphology with an average size of 13 nm and 12 nm (Fig. 1c and d), respectively. The inset HRTEM images show an interplanar distance of 0.35 nm, which can be assigned to the (111) plane of the cubic phase ZnS.

**Fig. 2:**
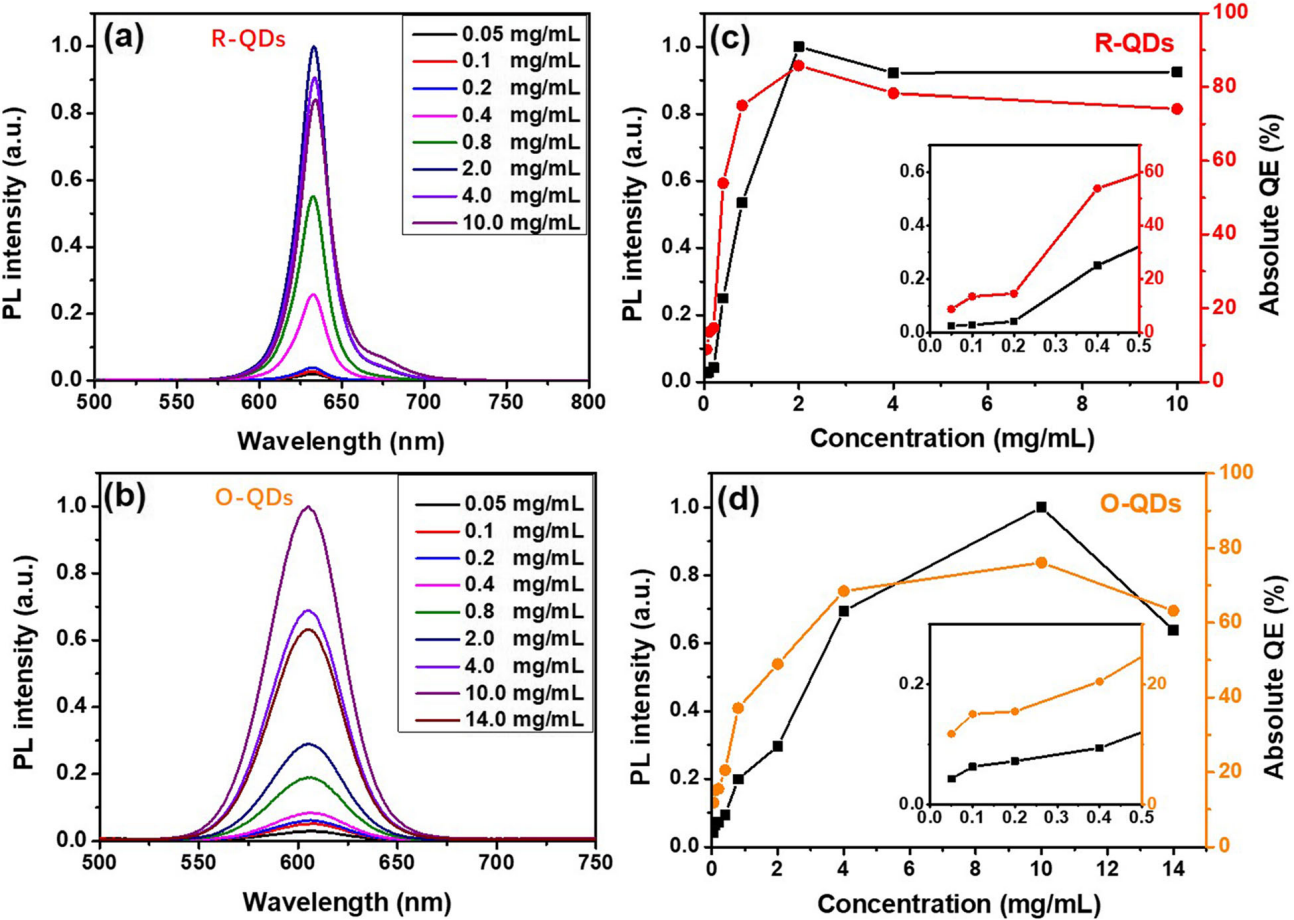
PL spectra of R-QD (**a**) and O-QD (**b**)-based silicone gel thin films. Corresponding PL intensity and QE of R-QD (**c**) and O-QD (**d**)-based silicone gel thin films with different concentrations of QDs

The optical properties of QDs silicone thin films made of the monochromic R-QDs and O-QDs with different concentrations are further tested under excitation by a 365 nm laser at 15.88 mW/cm^2^. Figure 2a and b show the concentration-dependent PL spectra of the QDs and their FWHM is almost constant. Figure 2c and d exhibit the PL intensity and absolute QE of monochromic QDs silicone thin films with different concentrations of QDs. With the rise of concentration, the PL intensity of R-QD silicone thin films increases until the QD concentration reaches 2 mg/mL and then decreases because of concentration quenching. Similar to the variation in PL intensity, the QE of the QDs reaches the highest value of about 85% at the same concentration. The PL intensity and QE of the O-QD silicone thin films present a similar concentration-dependent trend compared to those of the R-QD-based thin films. Differently, the PL intensity and QE of the O-QDs rise quickly till a QD concentration of 4 mg/mL and the maximum values are obtained at the concentration of 10 mg/mL. We infer that it is attributed to the larger Stoke’s shift of the O-QDs than the R-QDs. The maximum QE of the O-QD silicone thin film is about 76%, which is at the same QD concentration for the highest PL intensity.

Moreover, the 2 mg/mL R-QDs and 10 mg/mL O-QD-based silicone gel films also exhibit the highest PL intensity under different exciting power, as shown in Fig. S1 a and b, respectively. At the above two concentrations, the optical properties of the monochromic QDs are effectively preserved, which weakens the PL quenching caused by the host matrix effect [28, 29]. The study helps to find the suitable concentration of the monochromic QDs in silicone film.

**Fig. 3:**
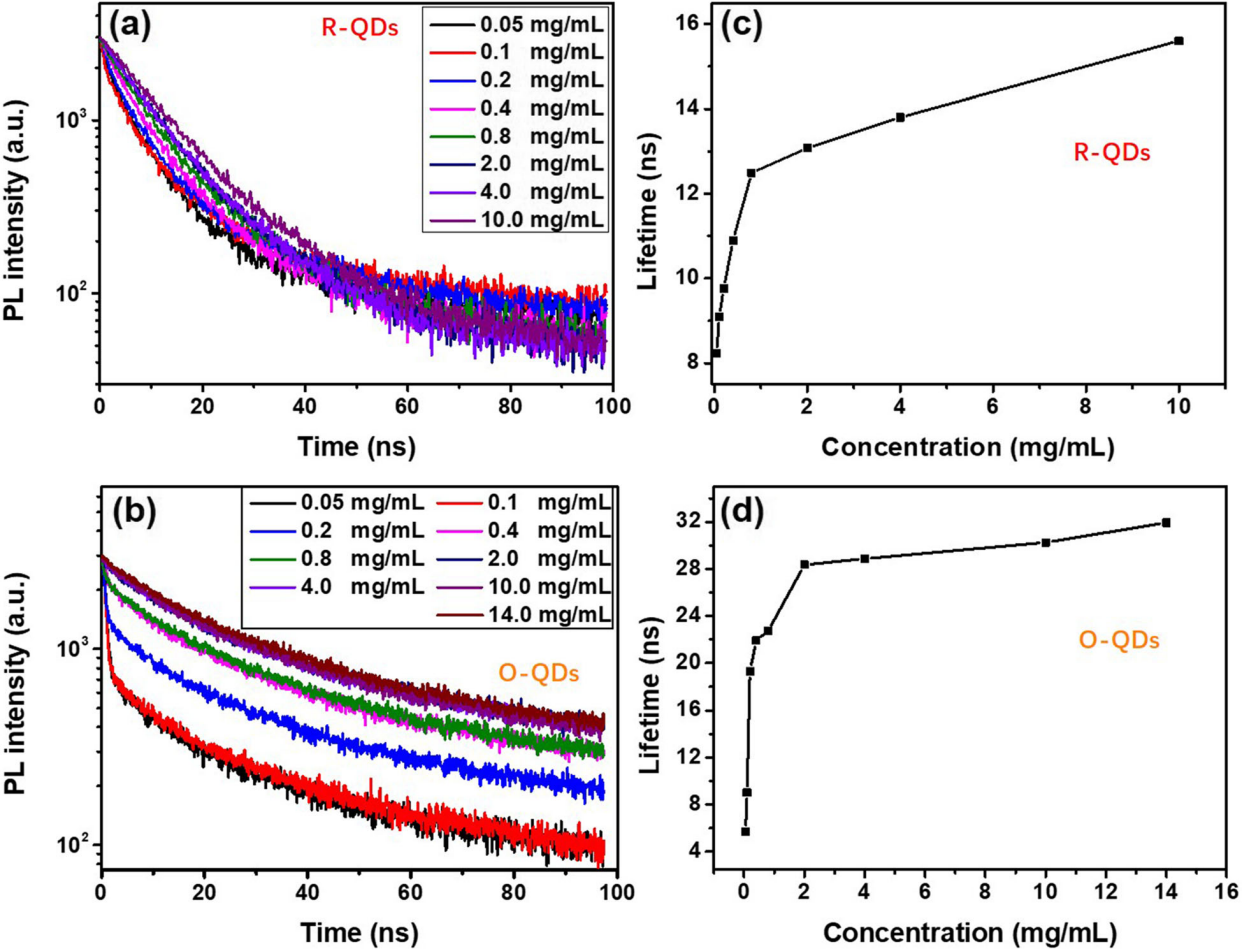
TRPL decay curves of R-QD (**a**) and O-QD (**b**)-based thin films. Lifetimes of R-QD (**c**) and O-QD (**d**)-based silicone gel thin films with different concentrations

To further investigate the concentration influence of QDs in the monochromic QD-based silicone thin films, the time-resolved PL (TRPL) spectra of the thin films with different concentrations are measured and the decay curves are depicted in Fig. 3. It is known that PL decay curves can be expressed with a multi-exponential function, as illustrated by Eq. 1 [30],

1 $$ I(t)=\sum \limits_{i=1}^n{A}_i{e}^{-t/{\tau}_i} $$

where *I*(*t*) is the PL intensity at time *t*, *A*_*i*_ and *τ*_*i*_ represent the relative amplitude and the excited state lifetime of each exponential component of PL decay, and *n* is the number of decay times. These decay curves, as shown in Fig. 3a and b, can be well fitted by a double exponential function according to Eq. 1.

The fitting parameters *A*_*i*_ and *τ*_*i*_ are listed in Table S1 and S2. The amplitude-weighted lifetimes of the R-QDs and O-QDs are selected as their lifetimes (*τ*_ave_) for further investigation. The lifetime can be calculated from the following Eq. 2 [31] and is listed in Table S1 and S2.

2 $$ {\tau}_{\mathrm{ave}}=\frac{A_1{\tau}_1+{A}_2{\tau}_2}{A_1+{A}_2} $$

Figure 3c and d show the lifetimes of the two monochromic QDs under different concentrations. Both lifetimes increase with the rise of concentrations and the rising rate becomes slower after 1 mg/mL for the R-QDs and 2 mg/mL for the O-QDs, respectively. It indicates that the rise of concentration reduces the distance among QDs thus enhances the energy transfer and self-absorption in the monochromic QDs [32, 33]. Meanwhile, the increment of the lifetime in O-QDs is more obvious than that in R-QDs, suggesting more energy transfer in O-QDs. However, it appears that the energy transfer does not induce fluorescent quenching of the QDs at low concentration. On the contrary, it may have a positive effect on the PL intensity and QEs, as previous shown in Fig. 2.

**Fig. 4:**
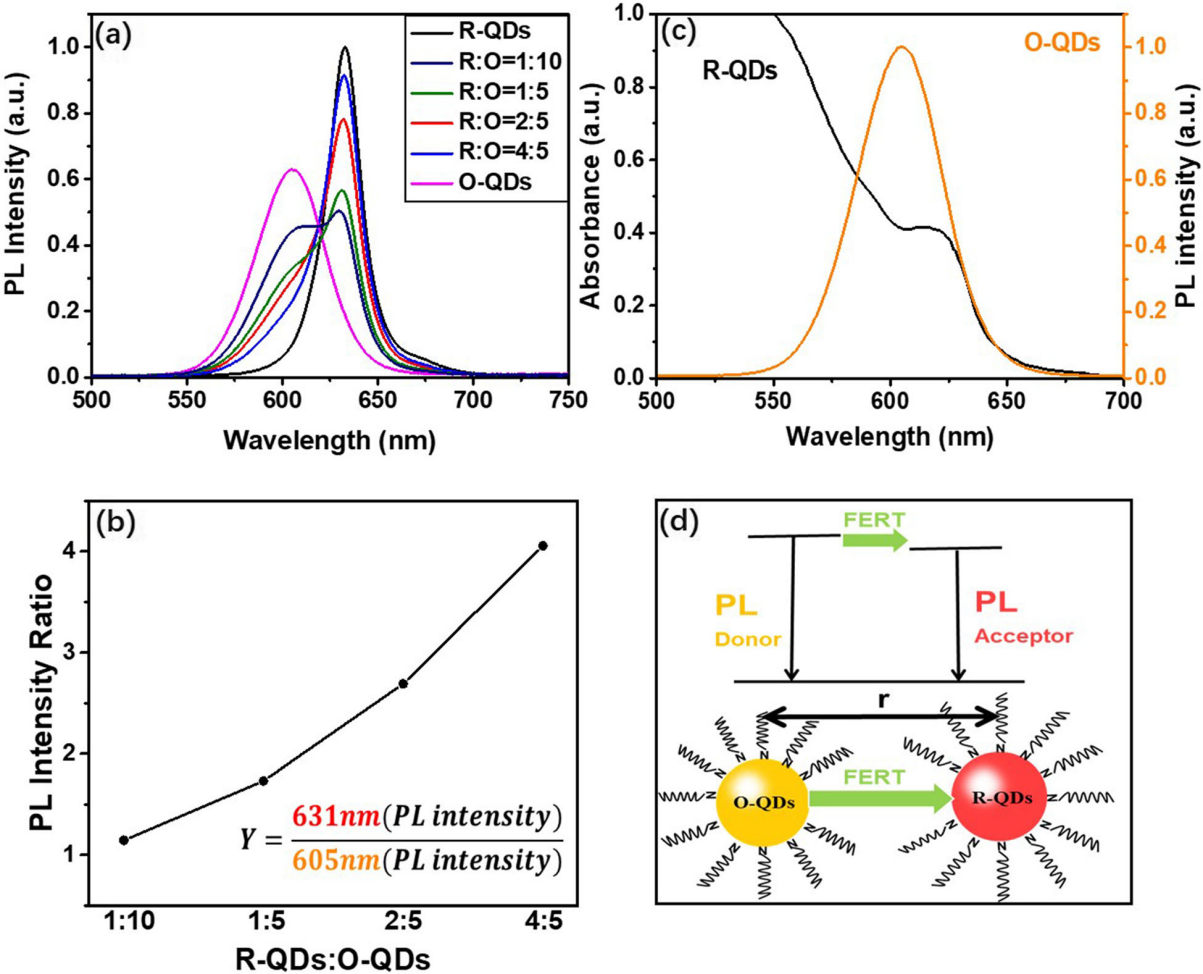
PL spectra of composite-QD silicone thin films with different R-QDs to O-QDs ratio (**a**) and the composite-QD PL peak intensity ratio (**b**). The overlap of the R-QD absorption spectrum and O-QD emission spectrum (**c**). Schematic diagram of energy transfer in composite-QDs (**d**)

The optical properties of composite-QDs with different weight ratios of R-QDs to O-QDs were further studied. The PL spectra of the composite-QD thin films are shown in Fig. 4a. Based on the spectra, the composite-QD PL peak intensity ratio of 631:605 (nm) is extracted in Fig. 4b. The peak intensity ratio presents a rising increment with the R-QD percentage, which suggests the energy transfer from O-QDs to R-QDs. Figure 4c exhibits the overlap between the R-QD absorption spectrum and the O-QD emission spectrum. It suggests a high chance of FRET process, in which the O-QDs acts as the donor and R-QDs as the acceptor (shown in Fig. 4d).

**Fig. 5:**
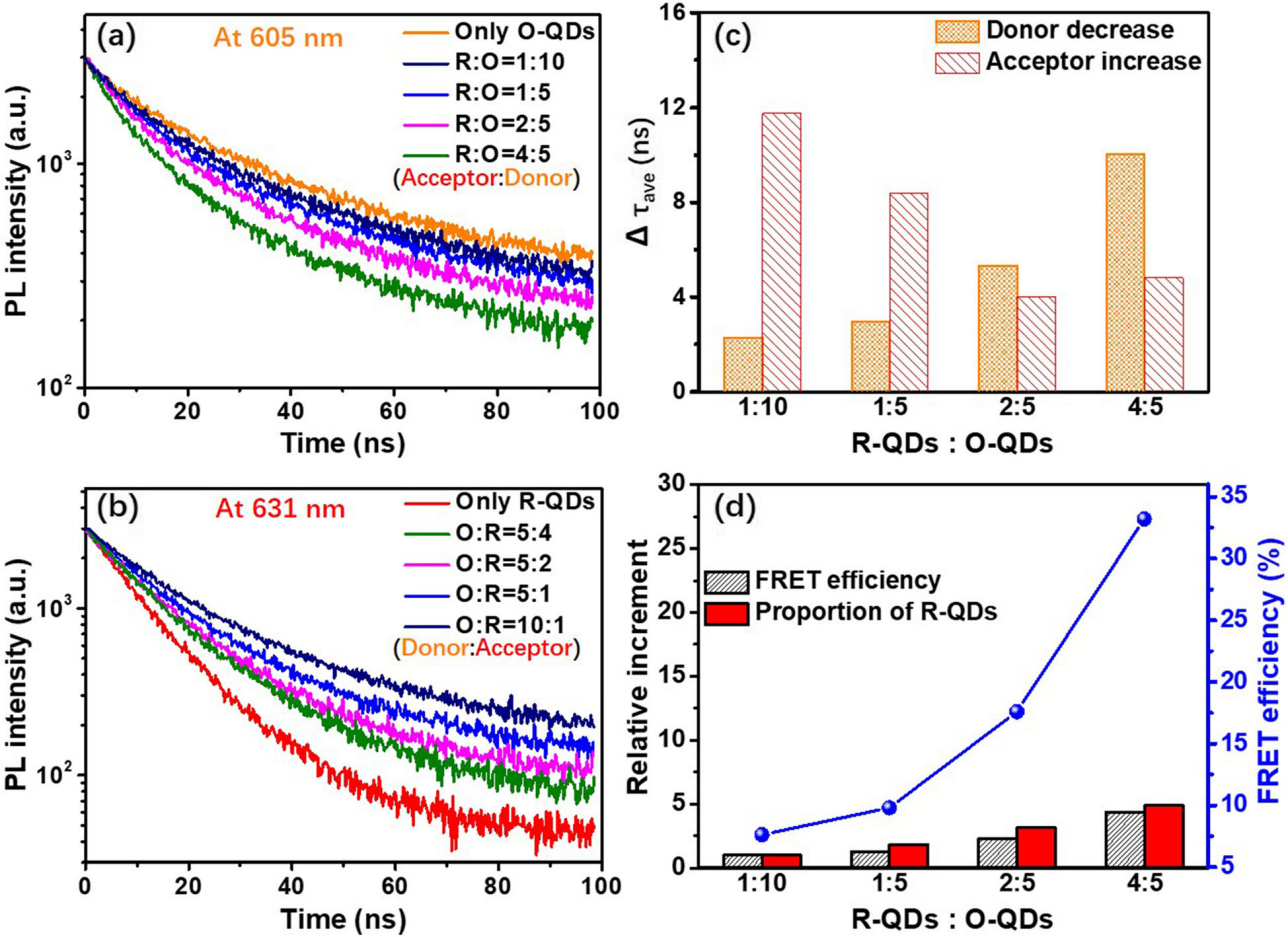
TRPL decay curves of composite-QD thin films with different ratios of R-QDs to O-QDs at donor peak emission wavelength (**a**) and acceptor peak emission wavelength (**b**). The variation of donor decrement lifetime and acceptor increment lifetime (**c**). The comparison of relative increment between in FRET efficiency and R-QDs proportion, and the calculated FRET efficiency under the different ratios of R-QDs to O-QDs (**d**)

Further study focuses on the FRET process in composite-QDs. Figure 5a presents the effect of R-QDs (acceptor) on the emission kinetics of the O-QDs (donor). The TRPL intensity decreases with the rise of acceptor in the film sample (analyzed at peak donor emission wavelength 605 nm). Figure 5b presents the effect of O-QDs (donor) on the emission kinetics of the R-QDs (acceptor). On the contrary, the TRPL intensity increases with the rise of the donor in the film sample (analyzed at peak acceptor emission wavelength 631 nm). The decay curves in Fig. 5a and b can be fitted with the 2 exponentials, and the detailed amplitudes, lifetime components, and amplitude-weighted lifetimes of the QDs were listed in Table S3. The lifetime of the O-QD sample was found to be 30.25 ns. When the acceptor R-QDs are introduced, the lifetime of the donor O-QDs decreases (Table S3) owing to the intervention of the energy-transferring channel. The lifetime of the donor becomes shorter with the rise of the acceptor concentration. On the contrary, the lifetime of the R-QD sample was found to be 13.08 ns. When the donor O-QDs are introduced, the acceptor R-QDs present an increase in lifetime as a result of energy feeding (Table S3) [34]. The calculated results are shown in Fig. 5c, which clearly demonstrates the phenomena.

The FRET process is also investigated by energy transfer efficiency. The efficiency of FRET can be calculated according to lifetime as illustrated by Eq. 3.

3 $$ E=1-\frac{\tau_{DA}}{\tau_D} $$

where *τ*_*DA*_ is the donor fluorescence lifetime in the presence of the acceptor, *τ*_*D*_ is the donor fluorescence lifetime in the absence of acceptor [26]. It shows that *τ*_*DA*_ is inversely proportional to the energy transfer efficiency. Therefore, as the acceptor-donor ratio increases, the *τ*_*DA*_ becomes shorter and the energy transfer efficiency increases. Larger energy transfer efficiency reflects higher impact on fluorescence. We further analyze the FRET efficiency of composite-QDs in Fig. 5a. The calculated results are listed in Table 1 and the efficiency reaches 33.2% with the highest proportion of acceptor. Meanwhile, Fig. 5d exhibits the change of FRET efficiency under different ratios of donor to acceptor. The FRET efficiency increases with the rise of R-QDs (acceptor) in the composite-QDs and the increment rate of the efficiency is close to that of the R-QDs. It indicates that the increment of energy transfer is sensitive to the increment of the acceptor.

**Table 1:** FRET efficiency of composite-QD silicone thin films with different ratios of acceptor to donor

R-QDs:O-QDs (acceptor:donor)	τ_DA_	E
Only O-QDs	30.25 (τ_D_)	--
1:10	27.96	7.6%
1:5	27.29	9.8%
2:5	24.93	17.6%
4:5	20.2	33.2%

**Fig. 6:**
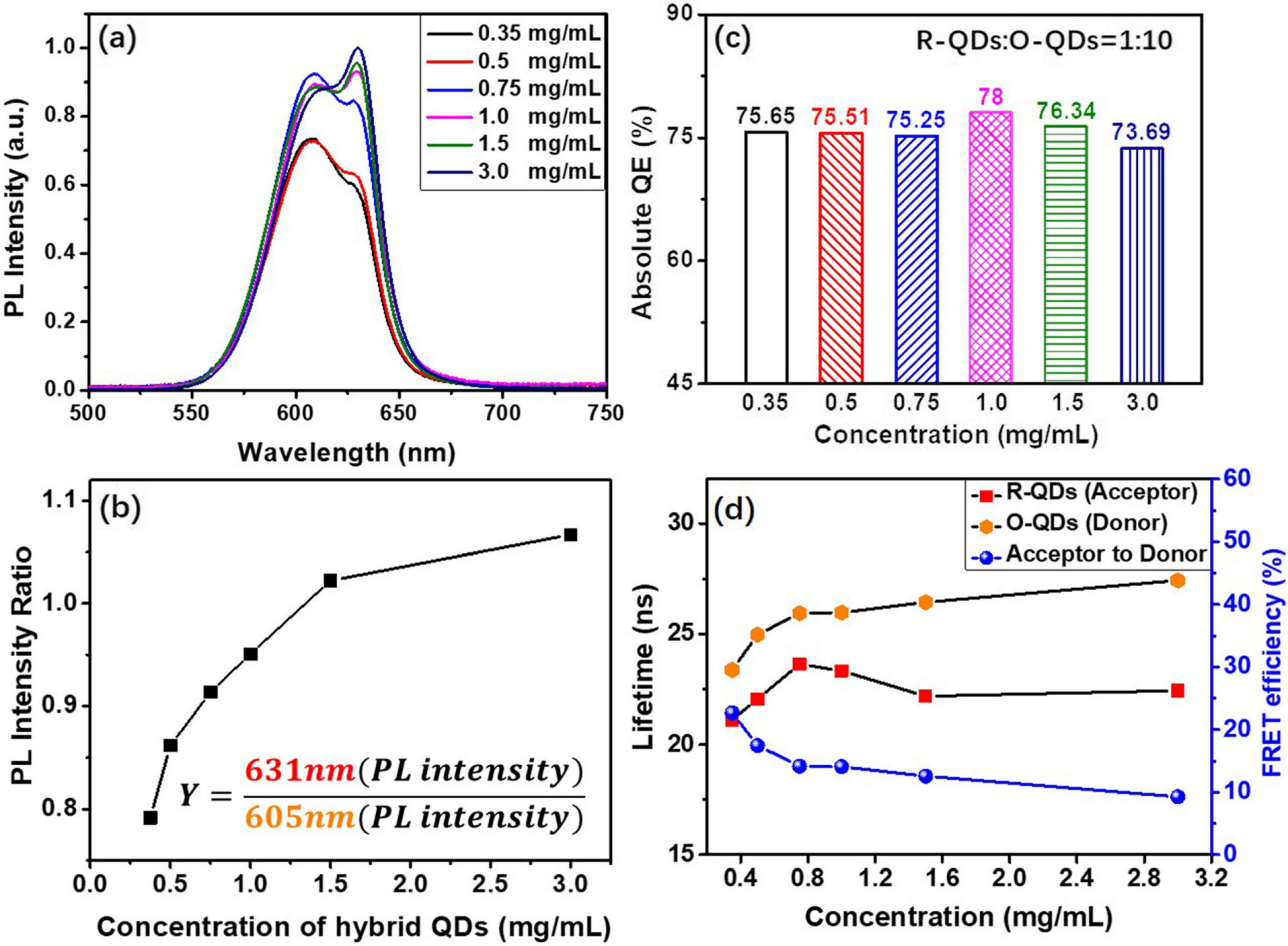
PL spectra of as-prepared composite-QD silicone thin films with different concentrations (**a**) and their PL peak intensity ratios (**b**). QE of the as-prepared composite-QD silicone thin films (**c**). Lifetime of donor (orange dots) or acceptor (red dots), and FRET efficiency (blue dots) under different concentration of composite-QDs (**d**)

As the best continuity spectrum for LED lighting in orange-red light, the composite-QDs with the 1:10 weight ratio of R-QDs to O-QDs is selected for further study. Figure 6a exhibits the PL spectra of the composite-QD silicone thin films with different concentrations of composite-QDs at the same weight ratio of R-QDs to O-QDs (R:O = 1:10). Besides the increase of the overall PL intensity, the proportion of the red-light (631 nm) also increases obviously with the rise of the QD concentration, as shown in Fig. 6b. This phenomenon can be attributed to the enhanced FRET with increasing QD concentration. Moreover, the rising rate of the red light becomes slower at higher QD concentration. This could be due to the saturation of the energy transfer (ET) among QDs. However, the absolute QEs of the QD silicone composite thin films exhibit a less than 5% change with different concentrations of composite-QDs, as shown in Fig. 6c. It appears that 1.0–1.5 mg/mL is the most favorable QD concentration for composite-QDs in application, which ensures high QEs with a low spectrum variation.

The TRPL decay curves of the different composite-QD concentration thin films are shown in Fig. S2. Table S4 lists the amplitudes, lifetime components, and amplitude-weighted lifetimes for the composite-QDs. Their FRET efficiencies are calculated and shown in Table S5. Furthermore, the changes in lifetimes and FRET efficiency with concentration are clearly shown in Fig. 6d. In detail, the FRET efficiency exhibits a decrement trend from 22 to 9% with the rise of concentration. Meanwhile, the lifetime recorded at the emission wavelength of the donor O-QDs increases with increasing concentration (orange dots in Fig. 6d). This is similar to the concentration-dependent lifetime of the pure O-QD samples shown in Fig. 3. It suggests the existence of the combined effect of FRET and self-absorption (like the monochrome QDs). With the rise of concentration, the enhanced self-absorption leads to the increment of the *τ*_*DA*_ (the donor fluorescence lifetime in the presence of the acceptor, as shown in Fig. 6d, orange dots), suggesting the inhibition of the FRET between composite-QDs (blue dots in Fig. 6d). At the acceptor R-QD emission wavelength, the reduced FRET efficiency results in smaller increment of the lifetime in high concentration (red dots in Fig. 6d). As a result, the composite-QDs show relative weak concentration-dependent lifetimes and could maintain a stable QE, which benefits the application of composite-QDs in LED applications.

**Fig. 7:**
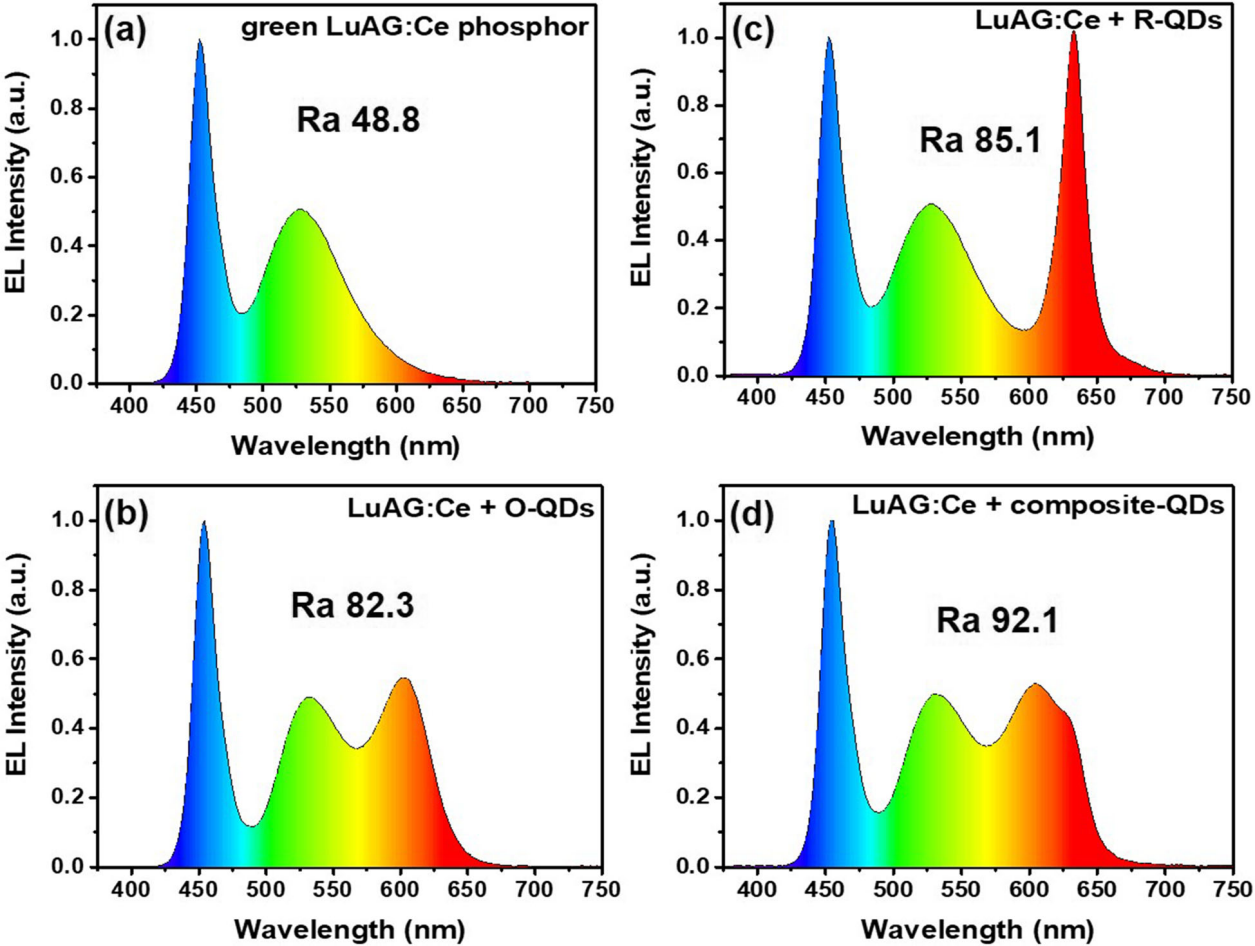
EL spectra of WLEDs packaged with green LuAG:Ce phosphor only (**a**), LuAG:Ce+R-QDs (**b**), LuAG:Ce+O-QDs (**c**), and LuAG:Ce + composite-QDs (d)

To study the light compensation effect of the composite-QDs in lighting application, WLEDs are fabricated by mixing green-emitting LuAG:Ce phosphor and O-QDs, R-QDs, or composite-QDs (R:O = 1:10) and packaging the mixture on top of 450 nm emitting GaN chips. Under a driving current of 40 mA, the excitation-luminescence (EL) spectra of the as-prepared WLEDs are illustrated in Fig. 7. The correlated color temperature (CCT) and color coordinates of the WLEDs are shown in Fig. S3 and Table S6. The four WLEDs have almost the same spectra in the blue-green light region but different in the orange-red light region. Besides, the LuAG:Ce (only)-based WLED shows the lowest color rendering index (CRI) of 48.8 due to the loss of red-orange light region. On the contrary, the composite-QD-based WLED exhibits a broader and flat spectrum in the orange-red light region and the highest CRI of 92.1. Compared to the composite-QDs, the LuAG:Ce (only) and R-QD-based WLEDs present obvious light gap in the orange light region and show great differences in CCT and color coordinates. Although the O-QD-based WLED has the similar CCT and color coordinates with the composite-QD-based WLED, it lacks the red light and thus presents a much lower CRI than that of the composite-QDs. It indicates the promising ability of composite-QDs in enhancing the color quality of WLED.

To further evaluate the experimental results, the luminous efficacy of radiation (LER) was calculated according to the following formula:

4 $$ \mathrm{LER}=683\frac{lm}{W_{\mathrm{opt}}}\frac{\int V\left(\lambda \right)P\left(\lambda \right) d\lambda}{\int P\left(\lambda \right) d\lambda} $$

where 683 *lm*/*W*_opt_ is a normalization factor. *W*_opt_, *V*(*λ*), and *P*(*λ*) are optical power, the human eye sensitivity function, and the spectral power density of the light source, respectively [35, 36].

The LER results are summarized in Table S6 and similar to previous reports [37–39]. According to the results, the LER of the composite-QD-based WLED (sample d) is higher than that of the R-QDs one (sample c) and lower than the O-QDs one (sample b) for the reason that human eyes are more sensitive to the orange light than the red light.

## Conclusion

In summary, we prepared the composite orange-red QDs (composite-QDs) and studied their optical properties and the energy transfer dynamic in the composite-QDs for LED applications. Our study reveals that the concentration of the composite-QDs and the proportion of donor-QDs and receptor-QDs play an important role in the energy transfer efficiency and spectrum stability. Meanwhile, the self-absorption has a significant influence on the FRET between different monochromic QDs in the composite-QDs. The relative stable and high QE can be achieved by adjusting the donor to receptor ratio in the composite-QDs, which is meaningful to enhance the color quality of WLED by compensation of the light gap in the orange-red region. As a result, the WLED fabricated based on the composite-QDs exhibits a highly improved color quality and more natural light spectrum compared to the spectra of the monochromic QD-based WLEDs.

## Supplementary information

**Additional file 1: Fig. S1.** The PL intensity of different concentration R-QDs (a) and O-QDs (b) silicone gels thin films excited by a 405 nm laser under different power. **Table S1.** Values for TRPL characteristics of R-QDs. **Table S2.** Values for TRPL characteristics of O-QDs. **Table S3.** Amplitudes, Lifetime Components, and Amplitude-Weighted Lifetimes for the composite-QDs at donor or acceptor emission wavelength under different ratios. **Fig. S2**. TRPL decay curves of the different concentration composite-QDs thin films at acceptor peak emission wavelength (a) and donor peak emission wavelength (b). **Table S4.** Amplitudes, Lifetime Components, and Amplitude-Weighted Lifetimes for the composite-QDs at Donor or Acceptor Emission Wavelength under different concentrations. **Table S5.** FRET efficiency of composite-QDs under different concentrations. **Fig. S3.** CIE diagram of WLEDs packaged with green LuAG:Ce phosphor only (a), LuAG:Ce + O-QDs (b), LuAG:Ce + R-QDs (c), and LuAG:Ce + composite-QDs (d).under 40 mA. **Table S6.** Luminescent parameters of the fabricated WLEDs.

## Data Availability

The datasets generated during and/or analyzed during the current study are available from the corresponding authors on reasonable request.

## Abbreviations

QDs: Quantum dots
WLEDs: White light-emitting diodes
composite-QDs: composite orange-red QDs
O-QDs: CdSe/ZnS-based orange QDs
R-QDs: CdSe/ZnS-based red QDs
FWHM: Full width at half maximum
FRET: Fluorescence resonance energy transfer
LEDs: Light-emitting diodes
CRI: Color rendering index
QWLEDs: QD-based WLEDs
CCT: Correlated color temperature
QE: Quantum efficiency
ODE: 1-Octadecence
S: Sulfur
TOP: Trioctylphosphine
Cd(St)_2_: Cadmium stearate
Se: Selenium powder
Zn(Ac)_2_: Zinc acetate
PL: Photoluminescence
TEM: Transmission electron spectroscopy
TRPL: Time-resolved PL spectroscopy
EL: Excitation-luminescence
