# Supplementary figures and images for: Study on the Color Compensation Effect of Composite Orange-Red Quantum Dots in WLED Application
Source: Nanoscale Res Lett. 2020 May 24;15:118. doi: 10.1186/s11671-020-03350-9 (PMC7246285; doi:10.1186/s11671-020-03350-9)

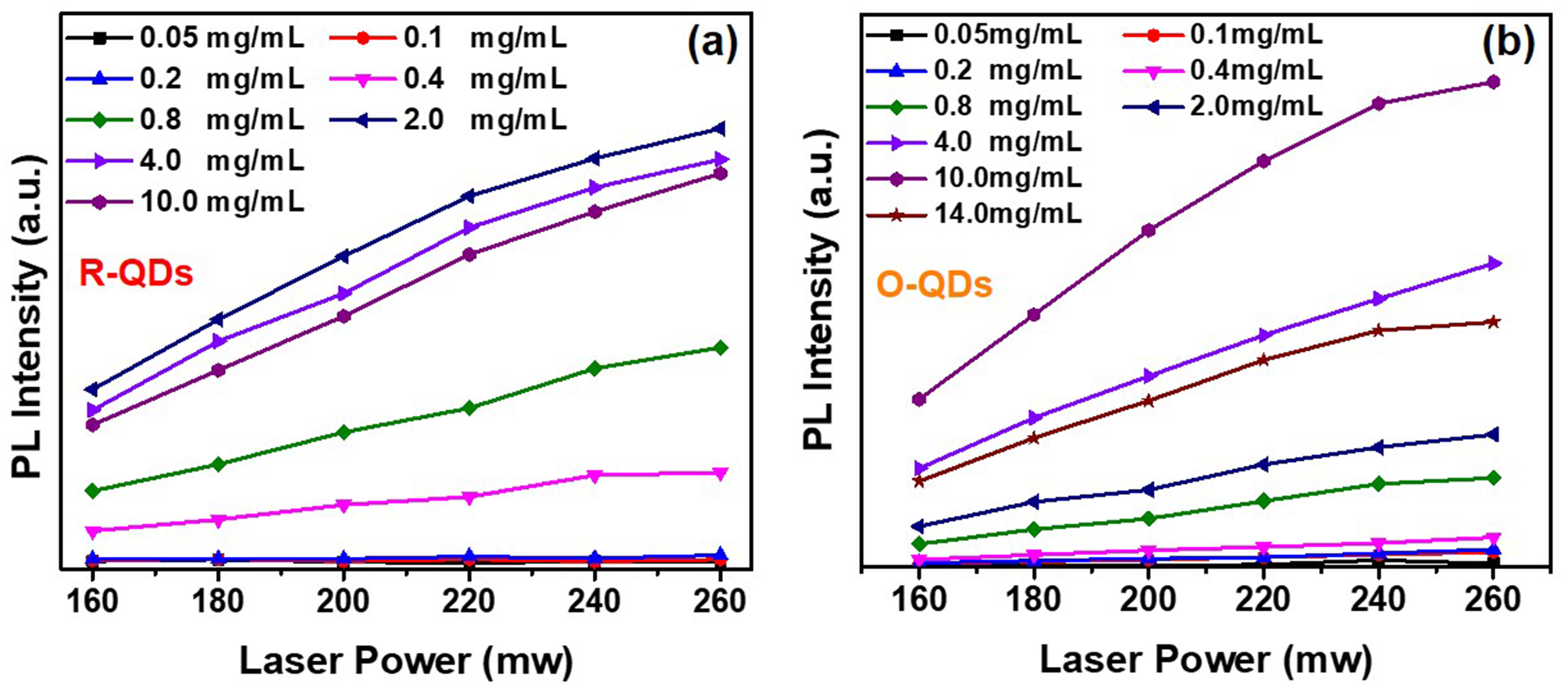

Supplement: Supplementary file 1 — Additional file 1: Fig. S1. The PL intensity of different concentration R-QDs (a) and O-QDs (b) silicone gels thin films excited by a 405 nm laser under different power. Table S1. Values for TRPL characteristics of R-QDs. Table S2. Values for TRPL characteristics of O-QDs. Table S3. Amplitudes, Lifetime Components, and Amplitude-Weighted Lifetimes for the composite-QDs at donor or acceptor emission wavelength under different ratios. Fig. S2. TRPL decay curves of the different concentration composite-QDs thin films at acceptor peak emission wavelength (a) and donor peak emission wavelength (b). Table S4. Amplitudes, Lifetime Components, and Amplitude-Weighted Lifetimes for the composite-QDs at Donor or Acceptor Emission Wavelength under different concentrations. Table S5. FRET efficiency of composite-QDs under different concentrations. Fig. S3. CIE diagram of WLEDs packaged with green LuAG:Ce phosphor only (a), LuAG:Ce + O-QDs (b), LuAG:Ce + R-QDs (c), and LuAG:Ce + composite-QDs (d).under 40 mA. Table S6. Luminescent parameters of the fabricated WLEDs. [file 11671_2020_3350_MOESM1_ESM.zip › Fig S1.tif]

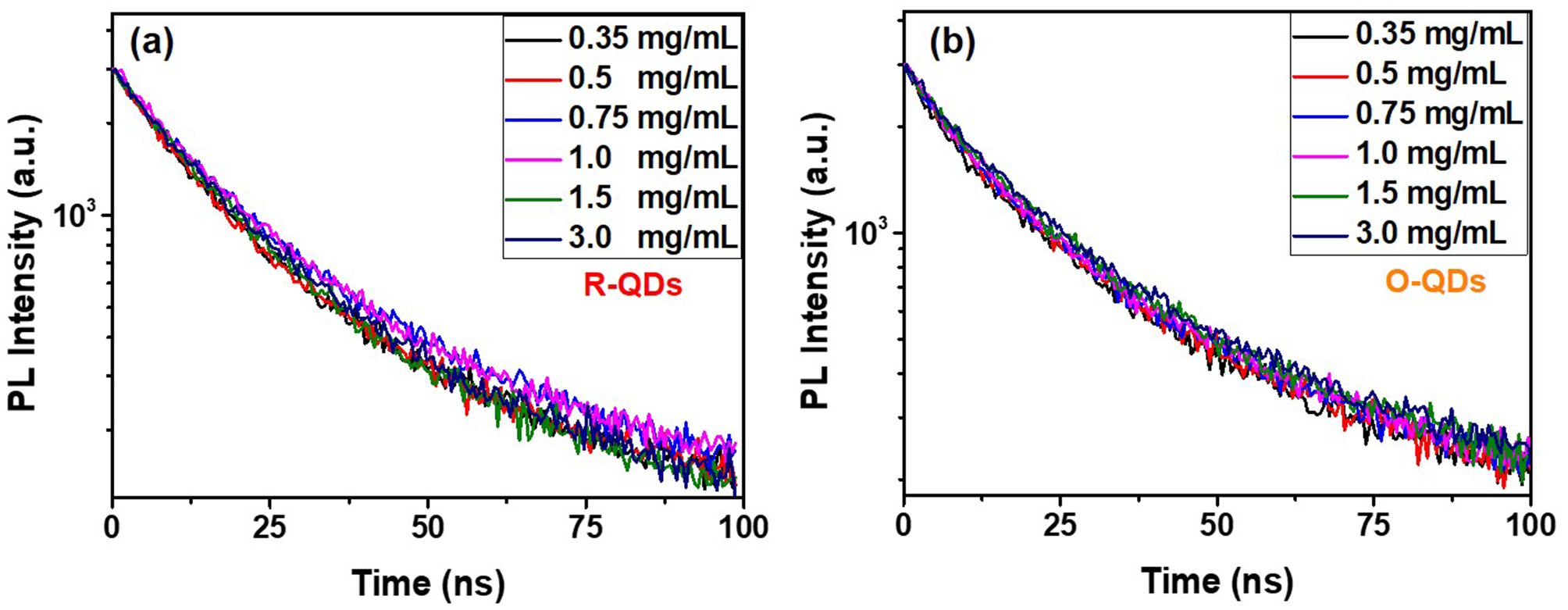

Supplement: Supplementary file 1 — Additional file 1: Fig. S1. The PL intensity of different concentration R-QDs (a) and O-QDs (b) silicone gels thin films excited by a 405 nm laser under different power. Table S1. Values for TRPL characteristics of R-QDs. Table S2. Values for TRPL characteristics of O-QDs. Table S3. Amplitudes, Lifetime Components, and Amplitude-Weighted Lifetimes for the composite-QDs at donor or acceptor emission wavelength under different ratios. Fig. S2. TRPL decay curves of the different concentration composite-QDs thin films at acceptor peak emission wavelength (a) and donor peak emission wavelength (b). Table S4. Amplitudes, Lifetime Components, and Amplitude-Weighted Lifetimes for the composite-QDs at Donor or Acceptor Emission Wavelength under different concentrations. Table S5. FRET efficiency of composite-QDs under different concentrations. Fig. S3. CIE diagram of WLEDs packaged with green LuAG:Ce phosphor only (a), LuAG:Ce + O-QDs (b), LuAG:Ce + R-QDs (c), and LuAG:Ce + composite-QDs (d).under 40 mA. Table S6. Luminescent parameters of the fabricated WLEDs. [file 11671_2020_3350_MOESM1_ESM.zip › Fig S2.tif]

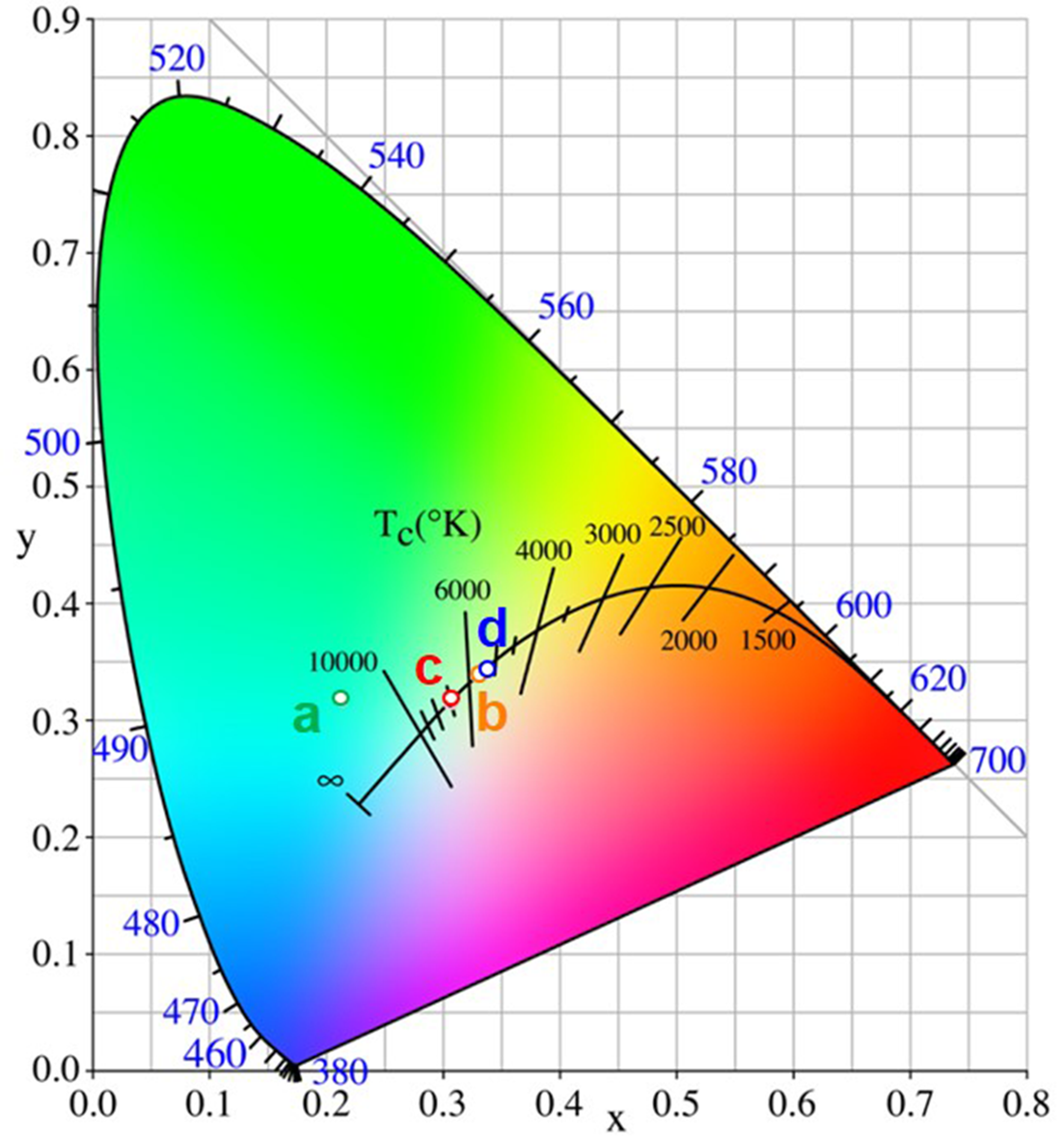

Supplement: Supplementary file 1 — Additional file 1: Fig. S1. The PL intensity of different concentration R-QDs (a) and O-QDs (b) silicone gels thin films excited by a 405 nm laser under different power. Table S1. Values for TRPL characteristics of R-QDs. Table S2. Values for TRPL characteristics of O-QDs. Table S3. Amplitudes, Lifetime Components, and Amplitude-Weighted Lifetimes for the composite-QDs at donor or acceptor emission wavelength under different ratios. Fig. S2. TRPL decay curves of the different concentration composite-QDs thin films at acceptor peak emission wavelength (a) and donor peak emission wavelength (b). Table S4. Amplitudes, Lifetime Components, and Amplitude-Weighted Lifetimes for the composite-QDs at Donor or Acceptor Emission Wavelength under different concentrations. Table S5. FRET efficiency of composite-QDs under different concentrations. Fig. S3. CIE diagram of WLEDs packaged with green LuAG:Ce phosphor only (a), LuAG:Ce + O-QDs (b), LuAG:Ce + R-QDs (c), and LuAG:Ce + composite-QDs (d).under 40 mA. Table S6. Luminescent parameters of the fabricated WLEDs. [file 11671_2020_3350_MOESM1_ESM.zip › Fig S3.tif]
