# Supplementary material for: Study on the Color Compensation Effect of Composite Orange-Red Quantum Dots in WLED Application
Source: Nanoscale Res Lett. 2020 May 24;15:118. doi: 10.1186/s11671-020-03350-9 (PMC7246285; doi:10.1186/s11671-020-03350-9)
Supplement: Supplementary file 1 — Additional file 1: Fig. S1. The PL intensity of different concentration R-QDs (a) and O-QDs (b) silicone gels thin films excited by a 405 nm laser under different power. Table S1. Values for TRPL characteristics of R-QDs. Table S2. Values for TRPL characteristics of O-QDs. Table S3. Amplitudes, Lifetime Components, and Amplitude-Weighted Lifetimes for the composite-QDs at donor or acceptor emission wavelength under different ratios. Fig. S2. TRPL decay curves of the different concentration composite-QDs thin films at acceptor peak emission wavelength (a) and donor peak emission wavelength (b). Table S4. Amplitudes, Lifetime Components, and Amplitude-Weighted Lifetimes for the composite-QDs at Donor or Acceptor Emission Wavelength under different concentrations. Table S5. FRET efficiency of composite-QDs under different concentrations. Fig. S3. CIE diagram of WLEDs packaged with green LuAG:Ce phosphor only (a), LuAG:Ce + O-QDs (b), LuAG:Ce + R-QDs (c), and LuAG:Ce + composite-QDs (d).under 40 mA. Table S6. Luminescent parameters of the fabricated WLEDs. [file 11671_2020_3350_MOESM1_ESM.zip › Supplementary Information.doc]

Supplementary Information

**Study on the Color-Compensation Effect of Composite Orange-Red Quantum Dots** **in WLED Application**

Xiaoyue Hu^1^, Yangyang Xie^1^, Chong Geng^1^, Shu Xu^1^*, Wengang Bi^1^*

^1^Tianjin Key Laboratory of Electronic Materials and Devices, School of Electronics and Information Engineering, Hebei University of Technology, 5340 Xiping Road, Tianjin, 300401, P. R. China

**Fig. S1** The PL intensity of different concentration R-QDs (a) and O-QDs (b) silicone gels thin films excited by a 405 nm laser under different power.

**Table S1** Values for TRPL characteristics of R-QDs.

| **R-QDs (mg/mL)** | **τ_1_ (ns)** | **A1** | **τ_2_ (ns)** | **A2** | **τ_ave_(ns) (amplitude)** |
| --- | --- | --- | --- | --- | --- |
| 0.05 | 1.81 | 1471.48 | 9.32 | 1698.07 | 8.23 |
| 0.1 | 1.56 | 1591.46 | 10.29 | 1517.21 | 9.09 |
| 0.2 | 2.87 | 1631.37 | 11.704 | 1416.60 | 9.76 |
| 0.4 | 5.33 | 1858.33 | 14.42 | 1081.55 | 10.90 |
| 0.8 | 7.19 | 2161.51 | 18.35 | 766.52 | 12.50 |
| 2 | 8.84 | 2342.99 | 21.50 | 484.01 | 13.08 |
| 4 | 9.64 | 2390.43 | 24.01 | 301.93 | 14.02 |
| 10 | 10.84 | 2456.77 | 29.95 | 295.29 | 15.60 |

**Table S2** Values for TRPL characteristics of O-QDs.

| **O-QDs (mg/mL)** | **τ_1_ (ns)** | **A1** | **τ_2_ (ns)** | **A2** | **τ_ave_(ns) (amplitude)** |
| --- | --- | --- | --- | --- | --- |
| 0.05 | 0.93 | 47511.29 | 21.72 | 605.26 | 5.70 |
| 0.1 | 0.81 | 26832.91 | 22.09 | 620.08 | 9.02 |
| 0.2 | 0.76 | 3848.85 | 21.61 | 1095.75 | 19.32 |
| 0.4 | 1.58 | 1144.80 | 22.87 | 1664.64 | 21.90 |
| 0.8 | 1.42 | 923.34 | 23.42 | 1747.17 | 22.73 |
| 2 | 7.38 | 762.52 | 31.11 | 1736.35 | 28.87 |
| 4 | 7.32 | 721.85 | 30.39 | 1787.94 | 28.34 |
| 10 | 8.81 | 889.29 | 33.28 | 1663.16 | 30.25 |
| 14 | 9.34 | 873.71 | 34.96 | 1737.89 | 31.92 |

**Table S3** Amplitudes, Lifetime Components, and Amplitude-Weighted Lifetimes for the composite-QDs at donor or acceptor emission wavelength under different ratios.

| **R-QDs:O-QDs(acceptor:donor)** | | **τ_1_ (ns)** | **A1** | **τ_2_ (ns)** | **A2** | **τ_ave_(ns) (amplitude)** |
| --- | --- | --- | --- | --- | --- | --- |
| 0:1 | 8.81 | | 889.29 | 33.28 | 1663.16 | 30.25 |
| 1:10 (605nm) | 8.50 | | 981.57 | 31.07 | 1681.02 | 27.96 |
| 1:5 (605nm) | 8.75 | | 1316.63 | 31.89 | 1455.28 | 27.29 |
| 2:5 (605nm) | 8.07 | | 1488.00 | 29.94 | 1348.15 | 24.93 |
| 4:5 (605nm) | 6.50 | | 1537.15 | 24.44 | 1320.03 | 20.20 |
| 1:0 | 8.84 | | 2342.99 | 21.50 | 484.01 | 13.08 |
| 4:5 (631nm) | 11.10 | | 2141.14 | 27.04 | 653.57 | 17.90 |
| 2:5 (631nm) | 9.14 | | 1273.83 | 20.06 | 1561.46 | 17.10 |
| 1:5 (631nm) | 11.98 | | 1943.19 | 29.93 | 869.71 | 21.45 |
| 1:10 (631nm) | 12.26 | | 1716.26 | 31.99 | 1156.47 | 24.84 |

**Fig. S2** TRPL decay curves of the different concentration composite-QDs thin films at acceptor peak emission wavelength (a) and donor peak emission wavelength (b).

**Table S4** Amplitudes, Lifetime Components, and Amplitude-Weighted Lifetimes for the composite-QDs at Donor or Acceptor Emission Wavelength under different concentrations.

| **sample** | **τ_1_ (ns)** | **A1** | **τ_2_ (ns)** | **A2** | **τ_ave_(ns) (amplitude)** |
| --- | --- | --- | --- | --- | --- |
| 0.35mg/ml (605nm) | 5.61 | 924.17 | 25.31 | 1874.90 | 23.37 |
| 0.5mg/ml (605nm) | 7.68 | 1123.07 | 28.07 | 1712.89 | 24.97 |
| 0.75mg/ml (605nm) | 8.69 | 1169.93 | 29.61 | 1614.30 | 25.94 |
| 1.0mg/ml (605nm) | 9.58 | 1078.28 | 29.37 | 1695.75 | 25.97 |
| 1.5mg/ml (605nm) | 8.93 | 1299.18 | 30.66 | 1566.63 | 26.44 |
| 3.0mg/ml (605nm) | 10.56 | 1234.22 | 31.86 | 1559.07 | 27.43 |
| 0.35mg/ml (631nm) | 9.97 | 1597.06 | 26.39 | 1277.49 | 21.12 |
| 0.5mg/ml (631nm) | 10.37 | 1793.23 | 28.84 | 1109.74 | 22.05 |
| 0.75mg/ml (631nm) | 10.49 | 1405.61 | 28.27 | 1479.24 | 23.64 |
| 1.0mg/ml (631nm) | 13.00 | 1959.49 | 33.05 | 818.79 | 23.33 |
| 1.5mg/ml (631nm) | 10.24 | 1337.05 | 26.12 | 1588.70 | 22.18 |
| 3.0mg/ml (631nm) | 11.38 | 1620.88 | 28.15 | 1265.25 | 22.43 |

**Table S****5** FRET efficiency of composite-QDs under different concentrations.

| **Composite-QDs (mg/mL)** | **τ_DA_**  **_9(_**  **_9_** | **E** |
| --- | --- | --- |
| Only O-QDs | 30.25(**τ_D_**) | -- |
| 0.35 | 23.37 | 22.7% |
| 0.5 | 24.97 | 17.5% |
| 0.75 | 25.94 | 14.2% |
| 1.0 | 25.97 | 14.1% |
| 1.5 | 26.44 | 12.6% |
| 3.0 | 27.43 | 9.3% |

**Fig. S3** CIE diagram of WLEDs packaged with green LuAG:Ce phosphor only (a), LuAG:Ce + O-QDs (b), LuAG:Ce + R-QDs (c), and LuAG:Ce + composite-QDs (d).under 40 mA.

**Table S6** Luminescent parameters of the fabricated WLEDs.

| **sample** | **Ra** | **CCT(K)** | **CIE(x,y)** | **LER (lm/ W_opt_)** |
| --- | --- | --- | --- | --- |
| a | 48.8 | 16637 | (0.2134,0.3173) | 316.88 |
| b | 82.3 | 5651 | (0.3291,0.3388) | 348.87 |
| c | 85.1 | 7010 | (0.3065,0.3148) | 275.60 |
| d | 92.1 | 5436 | (0.3362,0.3413) | 308.32 |
